# Cementation of a dual mobility cup in a well-fixed acetabular component- a reliable option in revision total hip arthroplasty?

**DOI:** 10.1186/s12891-021-04835-z

**Published:** 2021-11-24

**Authors:** Petri Bellova, Fiona Koch, Maik Stiehler, Albrecht Hartmann, Hagen Fritzsche, Klaus-Peter Günther, Jens Goronzy

**Affiliations:** Klinik für Orthopädie, Unfall- und Plastische Chirurgie, Universitätsklinikum Carl Gustav Carus an der Technischen Universität Dresden, Fetscherstr. 74, 01307 Dresden, Germany

**Keywords:** Dual mobility cup, “Cup-in-cup”, Double socket, Total hip arthroplasty, Revision

## Abstract

**Background:**

The “cup-in-cup” technique allows for revision of failed total hip arthroplasty (THA) when the cementless cup is well fixed. Furthermore, it can be used for liner wear or mechanical failure where liner replacement may be impossible or impractical. Recently, the “cup-in-cup” technique in combination with dual mobility cups (DMC) has drawn increased attention. Our aim was to report on the clinical and radiographic outcomes following this surgery.

**Methods:**

From 2015 to 2020, 33 patients treated with the DMC- “cup in cup” technique were retrospectively reviewed. Fourteen patients had died while 19 were available for the final follow-up (FU), of which 15 underwent both a radiograph and a FU visit, 2 underwent a radiograph only and 2 underwent a telephone interview only. Patient-related outcome measures included the HHS and the WOMAC. Radiographs were assessed for implant loosening and positioning. Primary endpoint was revision of any cause and secondary endpoint was loosening of the DMC at the latest FU. The survival analysis was conducted using the Kaplan-Meier method.

**Results:**

The mean age at surgery was 78.6 ± 7.1 (63–93) years and the mean surgery duration was 124.4 ± 52.0 (60–245) minutes. Recurrent dislocation (42.4%), periprosthetic fracture (39.4%) and polyethylene wear (6.1%) were the most frequent reasons for surgery. The mean FU duration (*n* = 19) was 28.5 ± 17.3 (3–64) months. The mean HHS score at FU was 59.4 ± 22.2 (29–91) and the mean WOMAC score was 59.7 ± 25.6 (15.6–93.8).

Two cups were revised due to instability and one revision was performed due to periprosthetic joint infection, accounting for an overall cup survival rate of 86.8% after a mean FU of 22.9 ± 18.0 (1.5–64.6) months. The survival rate free of loosening was 90.9% after a mean FU of 22.3 ± 18.5 (1.5–64.7) months.

**Conclusions:**

We found that the cementation of a DMC in a well-fixed cup is a promising short- to mid-term treatment addressing THA instability especially in elderly and frail patients, who benefit from a reduced operation time. Proper cementation technique, adequate cup positioning as well as selection of a sufficiently large DMC are crucial for treatment success. Longer FUs will be needed in the future in order to further prove the benefit of this technique.

## Introduction

Revision total hip arthroplasty (THA) may present a challenging endeavour as it can be associated with extensive approaches, bone and blood loss and technical challenges.

The double socket technique is a treatment method, which was initially described as the cementation of a polyethylene (PE) liner into a well-fixed shell [1]. This method allows for revision of failed THA in situations where the cementless cup is well-fixed and minimizes potential morbidity due to bone loss, intraoperative bleeding or prolonged operative time, especially in elderly and frail patients [2]. Apart from the treatment of instability, the double socket technique may also be used for liner wear or mechanical failure in cases where liner exchange might be impossible or impractical due to damaged locking mechanisms, non-availability of the liner or suboptimal positioning of the acetabular component.

Since the double socket technique with conventional PE liners has been associated with dislocation rates of up to 22% [1], especially if the reason for revision is instability, alternative combinations have been used, including constrained liners [3, 4] or dual mobility cups (DMCs) [5–7]. Generally, while constrained liners have been associated with high failure rates of up to 42% [8], DMCs have been associated with good functional results and low failure rates both in primary and in revision THA [9].

In contrast to conventional liners, DMCs have a lower dislocation risk due to their higher jumping distance [10]. Furthermore, in contrast to constrained liners, the mobile liner articulating both with the acetabular shell and the femoral head increases the range of motion eventually limiting the edge forces which may provoke early mechanical loosening in constrained liners [11].

The advantage of these common DMC characteristics was confirmed in two recent systematic literature reviews, where the application in THA revision was evaluated. Reina et al. [12] found a (re-)dislocation rate for DMCs in revision of only 2.2% when compared with 7.1% in the control group (conventional head sizes up to 40 mm) after a FU duration of 4.1 years, while Darrith et al. [9] observed a 2.2% dislocation rate along with a 96.6% survivorship after 5.4 years of FU. While there has been an increased use of DMCs in both primary and revision THA there is still a lack of long-term data.

Wegrzyn et al. first proved the biomechanical feasibility of the double socket technique in combination with DMCs [13]. The following clinical studies [5–7], while hampered by small patient collectives and short follow-ups (FU), displayed good radiographic and functional results. In addition to investigating rather small patient collectives, these previous studies have focused on the double socket or “cup-in-cup” technique in elective revision THA. Our collective, meanwhile, also included a significant amount of non-elective cases, such as periprosthetic fractures.

Therefore, our aim was to report on the clinical and radiographic outcomes of the “cup-in-cup” technique in combination with DMCs in a mixed cohort of elective and non-elective THA revision cases. The primary endpoint was revision of any cause while the secondary endpoint was loosening of the cup.

## Methods

**Fig. 1:**
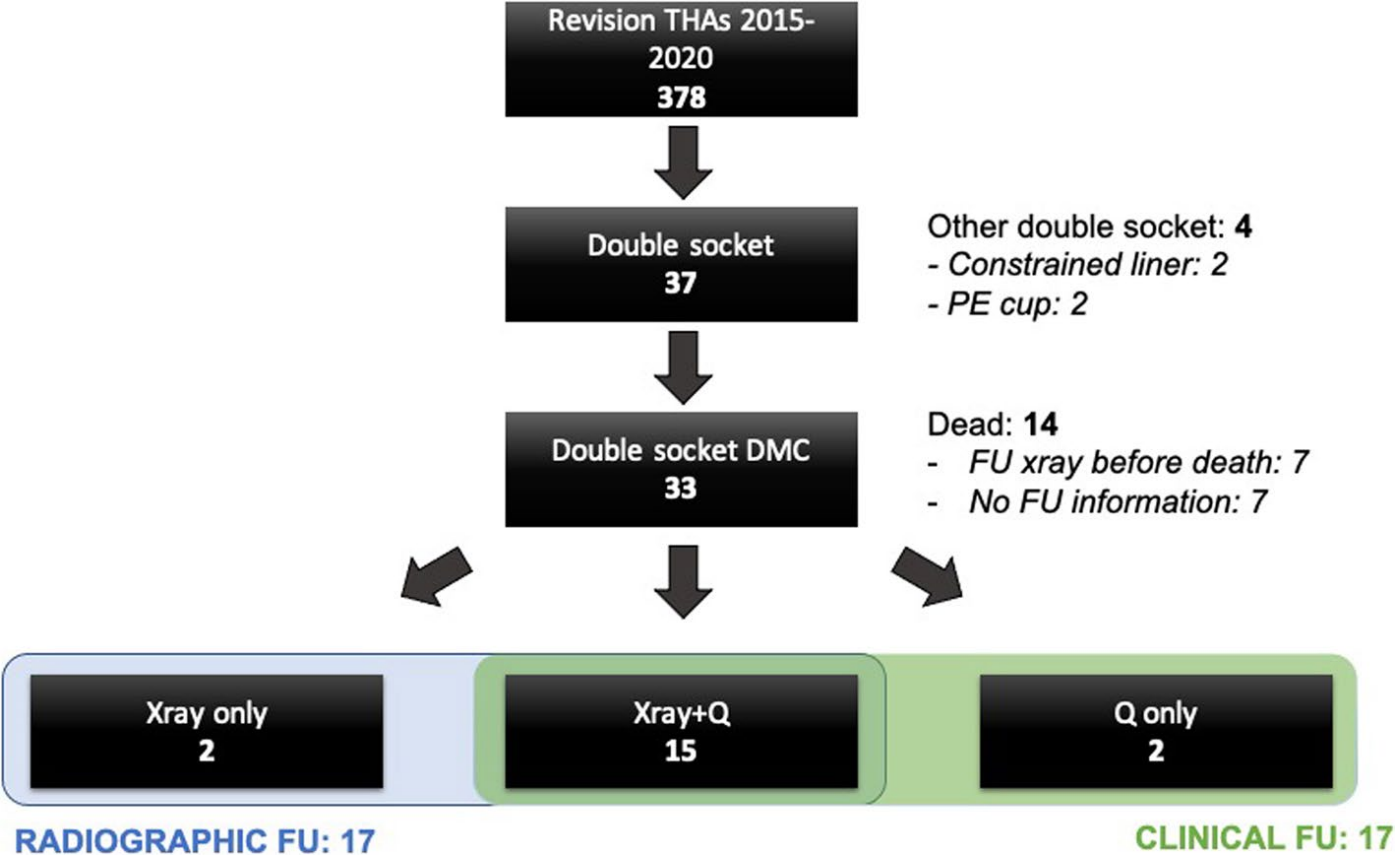
Flow chart of patient inclusion. DMC, dual mobility cup; FU, follow-up; PE, polyethylene; Q, questionnaire; THA, total hip arthroplasty

From January 2015 until March 2020, patients undergoing revision THA in our University Center were retrospectively reviewed. Patient’s informed consent and approval by the local ethics committee was obtained (institutional review board number BO-EK-253062020). Exclusion criteria were patients treated with alternative techniques, such as a complete cup exchange, the cementation of a PE cup in a porous shell or liner exchange of an existing DMC. Reviewing electronic records, we isolated 37 patients treated with the double socket technique, of which 33 had a DMC implanted into a well-fixed metal shell (Fig.1).

### Surgical technique

**Fig. 2:**
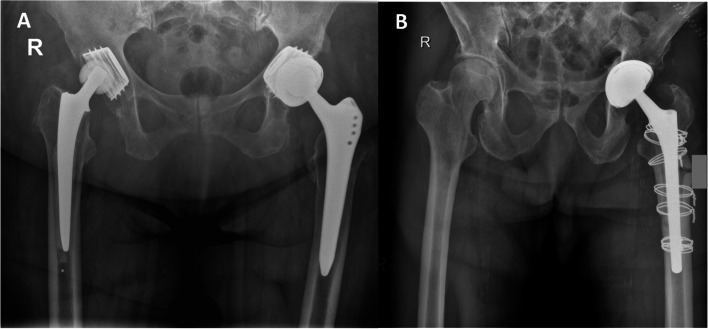
Different variations of double socket technique. (**A**) Double socket technique performed with the dual mobility cup (DMC) only partially embedded inside the well-fixed cup (left hip). (**B**) Double socket technique performed with the DMC fully embedded inside the well-fixed cup (left hip)

Revision THAs were performed by two experienced high-volume hip surgeons using a standard posterolateral approach in a lateral decubitus position. If a lateral approach had been used previously, the incision was extended posteriorly. If the previous approach was anterolateral, a new incision was made. After hip exposure and dislocation, the femoral head and the PE insert were removed. The metal shell as well as the femoral shaft were manually tested for stability. Careful debridement and pulsed irrigation of the joint were undertaken. Screws and other fixation points were removed in order to enhance the roughness of the primary implant. The DMC used was either Polarcup® (Smith and Nephew, London, United Kingdom; *n* = 29) or Ecofit 2 m® (implantcast, Buxtehude, Germany; *n* = 4). First, a trial component was used in order to determine the size that best fitted into the metal shell. If the cementless shell was large in size (≥54 mm), the DMC was fully embedded into the former cup. If the primary cup was smaller in size (< 54 mm), the DMC was partially embedded inside the metal shell leaving the DMC uncovered by the former implant at the edge (Fig.2). The minimum size of the primary cup used within our collective was 48 mm. Bone cement containing gentamicin (0.5 g per 40 g; Palacos G + R, Heraeus Medical, Germany) was mixed and then thickly applied and manually packed into the retained shell. Then, the cemented DMC was placed at the desired anteversion and inclination maintaining manual pressure with attention to avoid bottoming out of the component against the dome of the existing metal shell and held in place with a pusher until hardening of the cement. A trial femoral head and a dual mobility trial liner were used to evaluate intraoperative stability of the hip. Neck length was adjusted accordingly. The definite mobile components were inserted, the hip joint reduced, and the wound was closed layerwise.

### Baseline characteristics

Baseline characteristics including age, sex, operated side, reason for surgery, American Society of Anaesthesiologists (ASA) score and Charlson Comorbidity Index (CCI) [14] were derived from the patients’ electronic records.

Furthermore, operative records were assessed for information on the duration of surgery as a measure of the invasiveness of the intervention and the size of the acetabular components. The size of the metal shell was generally not included in the records and was therefore measured on available scaled radiographs. Elective patients with a heightened CRP underwent hip fluid aspiration for cell count and microbiological evaluation. Intraoperatively, histology as well as at least 5 microbiological samples, including sonication, were taken.

### Follow-up (FU)

Of the 33 patients included in this study, 14 had died before the final FU. Of the latter, 7 FU radiographs were available that were taken at least 6 weeks postoperatively. Radiographs taken prior to this were not considered. Of the 7 remaining patients, no information other than that they had deceased were available.

Of the 19 patients still alive at the final FU, all were contacted. If not contactable due to severe dementia, hearing difficulty or other reasons, relatives or guardians were contacted instead. Fifteen patients were available for both a FU examination and radiographs, two were available for a FU telephone interview only and two had a FU radiograph taken externally but were not available for the FU visit in our outpatient clinic. FU visits were conducted between October 2020 and March 2021. Thus, 17 patients were available for both radiographic assessment and clinical assessment at the final FU, respectively (Fig.1).

During the FU visit or telephone interview, the physician-based outcome score Harris Hip Score (HHS) [15] as well as the patient-related outcome measures (PROMs) Western Ontario and McMaster Universities Osteoarthritis Index (WOMAC) [16], University of California, Los Angeles (UCLA) score and Euroqol-5-Dimensions (EQ-5D) score [17] were determined using questionnaire forms. The UCLA was especially aimed at the functional outcome and the EQ-5D at life quality and potential depression.

**Fig. 3:**
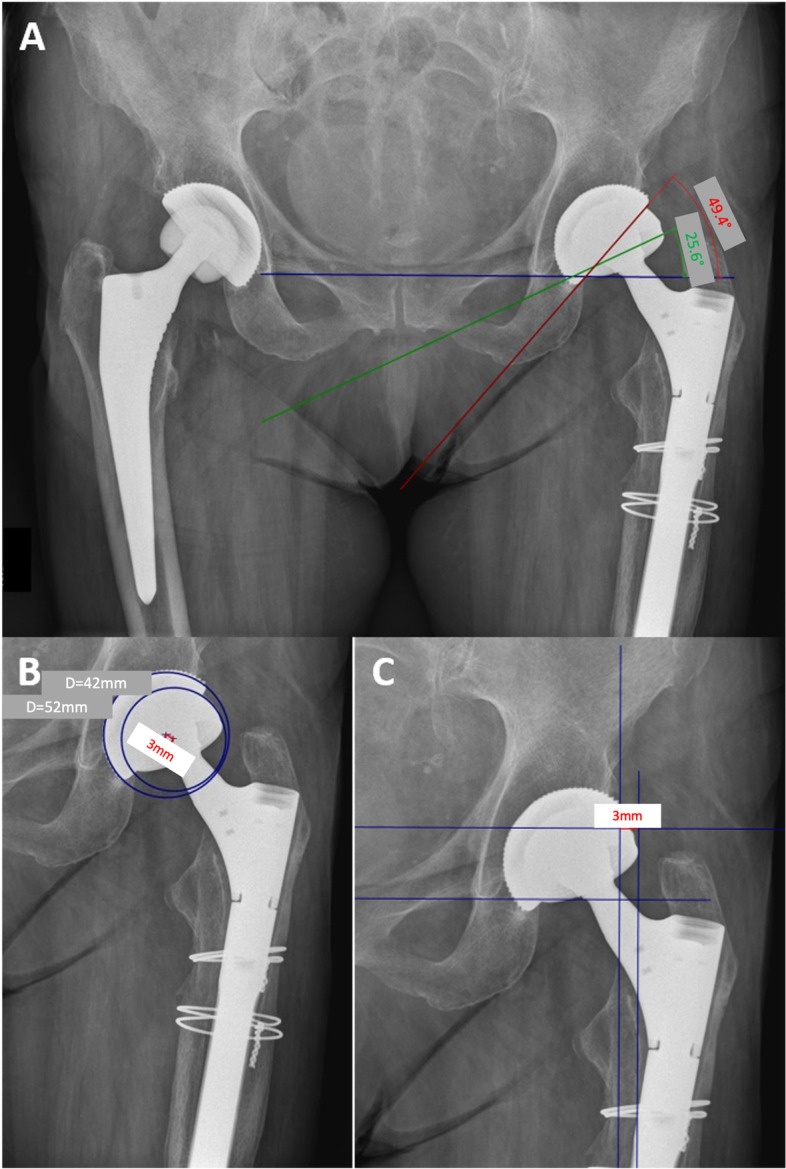
Radiographic measurements. (**A**) Measurement of inclination between well fixed cup (red line) and dual mobility cup (DMC; green line). The reference line is the connecting line between the two teardrops (blue line). (**B**) Measurement of the center of rotation distance (red connecting line) between the well-fixed cup and the DMC. (**C**) Measurement of the lateral edge distance between well-fixed cup and DMC (red line). Again, the connecting line between the lower aspects of Kohler’s teardrop figure (lower horizontal line) serves as the reference line

Native radiographs (pelvic and frog-leg hip radiographs) were performed in the supine position and assessed for signs of loosening, wear or periprosthetic fracture. The inclination of both the well-fixed cup and the new cup were determined. Furthermore, the center of rotation distance between the well-fixed cup and the new cup was determined. In addition, the lateral edge distance between the well-fixed cup and the new cup was determined (Fig.3). Finally, a qualitative assessment was undertaken evaluating whether 1) the DMC was fully embedded into the cup or not and 2) whether the DMC was placed in a more anteverted position as compared to the cup or not.

### Statistical analysis

Primary endpoint of the study was cup exchange or explantation due to any cause. For the survival analysis, all patients with an available FU (*n* = 26) were included. Secondary endpoint was loosening of the DMC (dissociation at the cement-implant interface) on the last available radiograph. For this analysis, all patients with an available FU radiograph (*n* = 24) were included. Both analyses were conducted using the Kaplan-Meier method.

Categorical variables were presented as counts with percentages, continuous variables as the mean with standard deviation and the range. The student t-test or the Fisher’s exact test were performed to determine statistical significance, as appropriate. The *p*-value was set at *p* < .05. All analysis was performed using SPSS V. 27 (IBM, CA, USA).

## Results

The baseline characteristics are shown in Table 1. Mean age at surgery was 78.6 ± 7.1 (63–93) years. Mean size of the well-fixed cup was 53.9 ± 3.2 (48–60), while the mean size of the DMC was 46.6 ± 2.5 (43–53). The mean difference between the two was 7.4 ± 2.3 (3–11).

**Table 1:** Baseline characteristics

baseline	
Age at surgery (y)	78.55 ± 7.05 (63–93)
ASA Score	2.76 ± .56 (1–3)
ASA 1	2/33 (6.1%)
ASA 2	4/33 (12.1%)
ASA 3	27/33 (81.8%)
CCI	5.15 ± 1.44 (3–9)
Surgery duration (min) isolated cup revision (*n* = 19) cup and stem revision (*n* = 14)	124.4 ± 52.0 (60–245) 91.9 ± 34.2 (60–166) 168.6 ± 37.2 (111–245)
Hospital stay (d)	13.6 ± 7.2 (5–34)
FU duration (n = 19; mo)	28.5 ± 17.3 (3–64)
**Size**	
*Well fixed cup (mm)*	53.9 ± 3.2 (48–60)
*DMC (mm)*	46.6 ± 2.5 (43–53)
δ (mm)	7.4 ± 2.3 (3–11)
sex (female)	21/33 (63.6%)
**diagnosis**	
*Recurrent dislocaton*	14 (42.4%)
*Periprosthetic fracture*	13 (39.4%)
*PE wear*	2 (6.1%)
*ALTR*	2 (6.1%)
*femoral loosening*	2 (6.1%)

*ALTR* adverse local tissue reaction; *ASA* American Society of Anaesthesiologists; *CCI* Charlson Comorbidity Index; d, days; *FU* follow-up; *min* minutes; *mm* millimeter; *mo* months; *PE* polyethylene; *SD* standard deviation; *y* years; δ difference

**Fig. 4:**
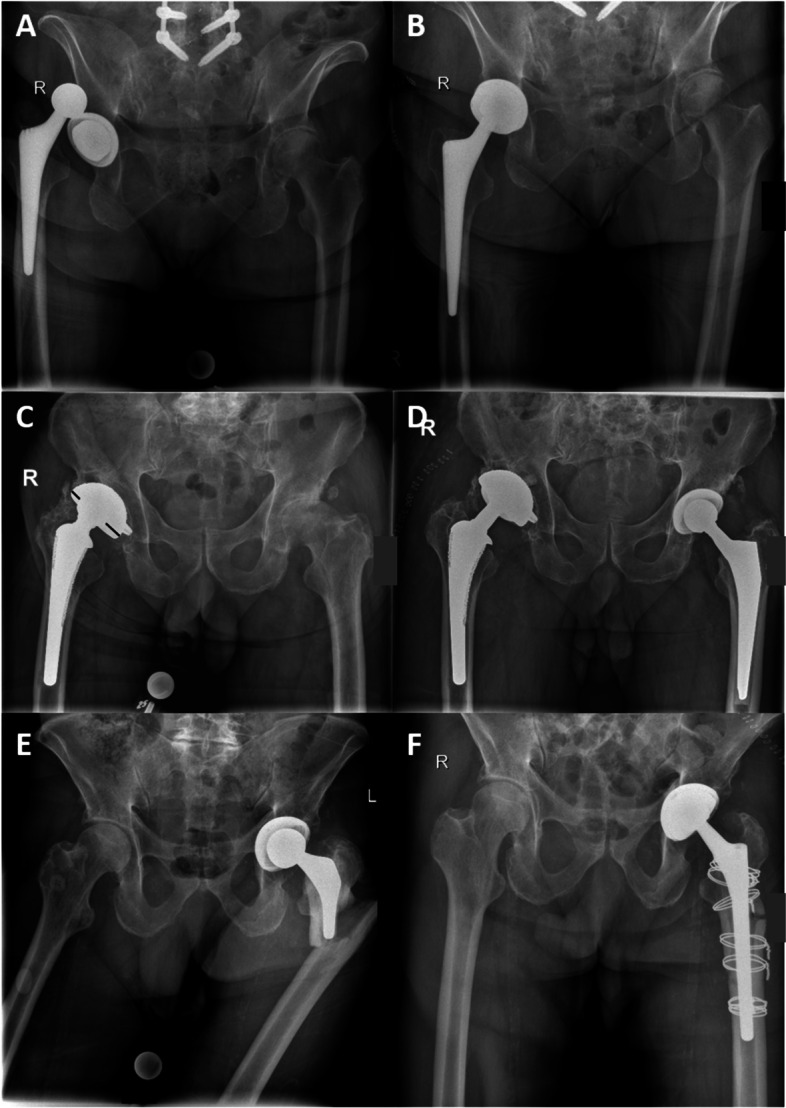
Indications leading to surgery with the “cup-in-cup” technique. (**A** + **B**) Recurrent dislocation; (**C** + **D**) polyethylene wear, as seen by the decentralized head within the cup (black lines indicating the asymmetry); (**E** + **F**) periprosthetic fracture necessitating both stem revision and cup revision with the “cup-in-cup” technique in order to prevent dislocation

The diagnoses leading to revision surgery are also displayed in Table 1 as well as in Fig.4.

The mean FU duration (*n* = 19) was 28.5 ± 17.3 (3–64) months at the latest FU. Of the 14 patients that had died before the final FU, 7 FU radiographs were available in our electronic patient records at a mean time of 7.9 ± 10.9 (1–32) months after surgery.

**Fig. 5:**
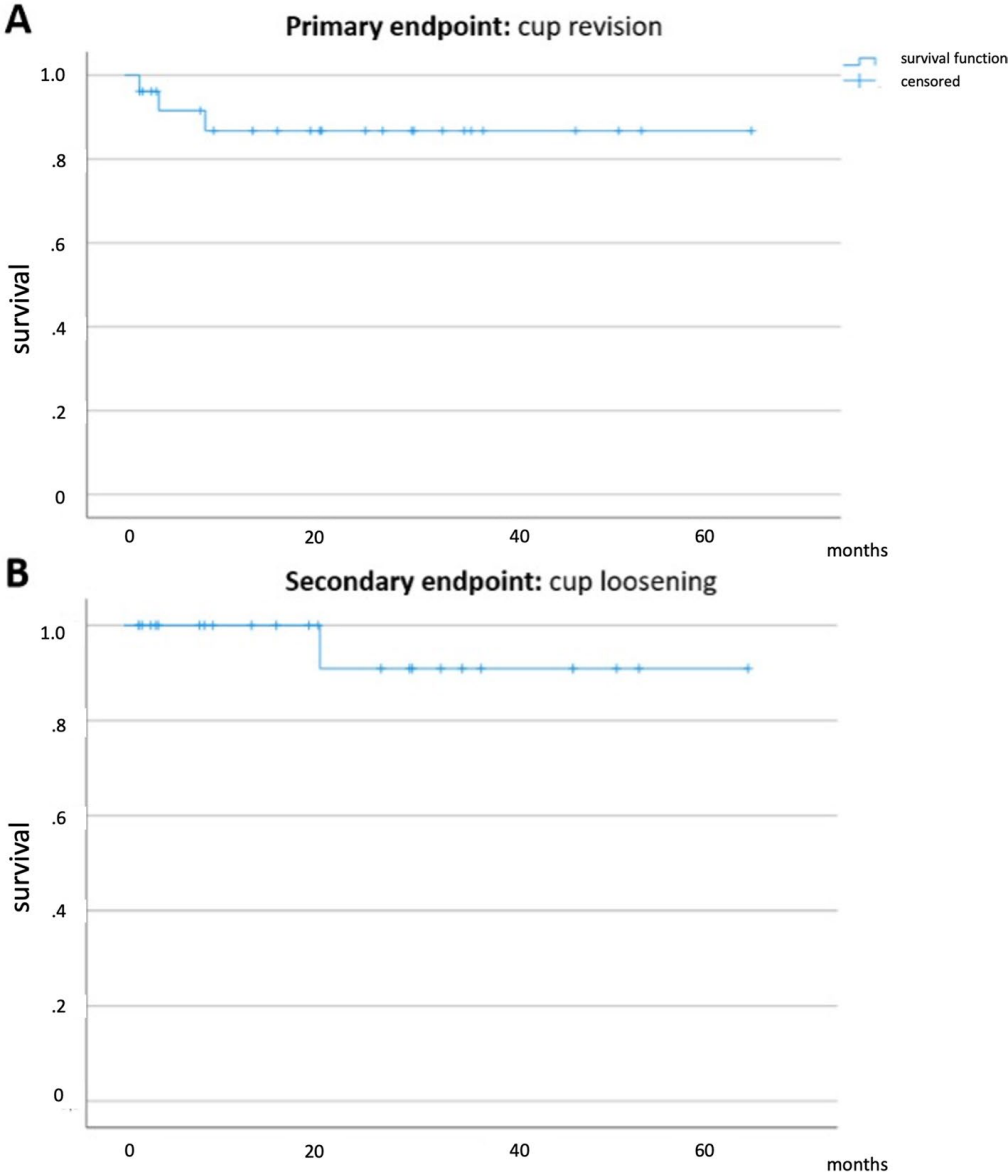
Kaplan Meier survival curve depicting primary endpoint “cup revision of any cause” (**A**) with a survival rate of 86.8% after a mean follow-up (FU) of 22.9 ± 18.0 (1.5–64.6) months and secondary endpoint “cup loosening” (**B**) with a survival rate of 90.9% after a mean FU of 22.3 ± 18.5 (1.5–64.7) months

Of the 26 patients with an available FU, two underwent cup revision due to recurrent dislocation while one patient underwent explantation of the whole prosthesis due to periprosthetic joint infection (PJI). Using the Kaplan Meier method, this accounted for an overall cup survival rate of 86.8% after a mean FU of 22.9 ± 18.0 (1.5–64.6) months (Fig.5).

**Fig. 6:**
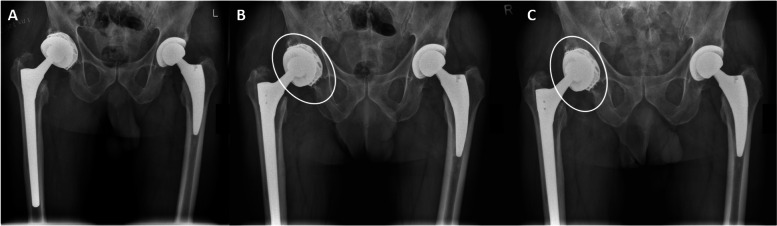
Postoperative radiographs (05/2018) (**A**), at 3 months postoperatively (**B**) and at the final follow-up (FU; 04/2020) (**C**) after “cup-in-cup” revision with a dual mobility cup (DMC). The cup position had already changed at the 3 month-FU (**B**) and had not further changed at the last FU (**C**), being at a much steeper position inside the well-fixed cup (white ellipses). This change of position was indicative of loosening of the DMC

Of the 24 patients with an available FU radiograph, there was one case of dissociation at the cement-cup interface (Fig.6) which accounted for a survival rate free of loosening of 90.9% after a mean FU of 22.3 ± 18.5 (1.5–64.7) months.

**Fig.7:**
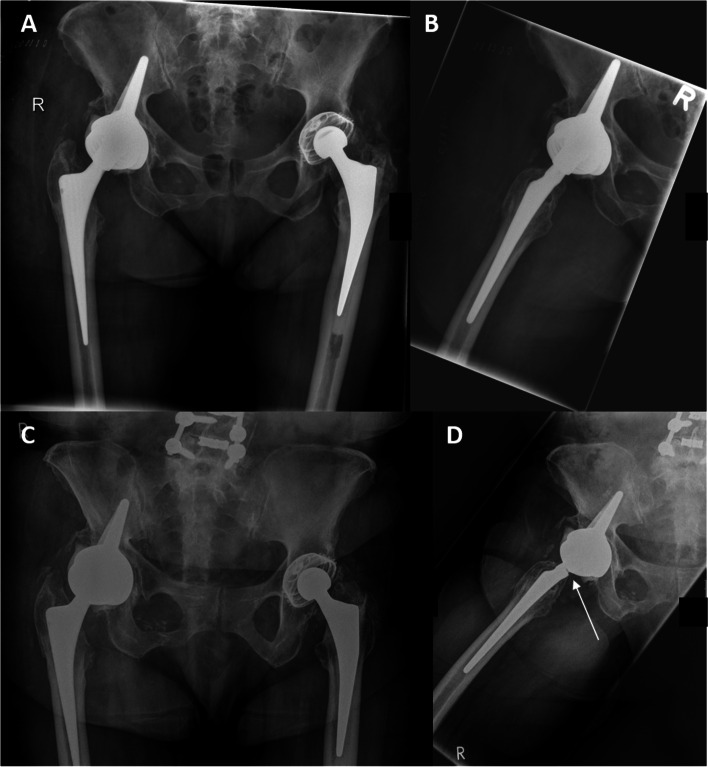
(**A** + **B**) Postoperative radiographs after double socket procedure with a dual mobility cup (DMC) implanted in a socket cup (09/2016). The taper shows no signs of damage at this stage (**B**). At FU (03/2020; **C** + **D**), the cup position had remained unchanged, however, signs of advanced taper corrosion were seen on the lateral radiograph (**D**), indicated by the groove at dorsal aspect of the cone (arrow), presumably due to taper impingement on the dorsal aspect of the DMC

The two patients that suffered a dislocation of the DMC following the “cup-in-cup” technique were revised with a constrained liner. An analysis of the potential causes of failure revealed that both dislocations occurred with a 43 mm-sized DMC. The explantation was due to PJI and was performed after one failed attempt of irrigation and debridement (I&D). The patient with the cement-implant dissociation at FU did not receive a cup revision since he was under chemotherapy due to an intracranial tumor and was still able to walk on crutches despite the loose DMC. At the latest FU, he was still alive and mobile. Apart from these cases, one patient suffered a periprosthetic femoral fracture during FU which was treated with open reduction and internal fixation (ORIF). Furthermore, one patient had signs of taper damage on the FU radiographs without reporting any hip-related symptoms (Fig.7).

The results of the PROMS and their respective subscores are depicted in Table 2.

**Table 2:** Results of patient-related outcome measures (PROMs)

Score		
HHS	*Pain*	26.9 ± 12.8 (10–44)
	*Function*	24.4 ± 11.1 (2–41)
	*AoD*	4.0 ± .0 (4–4)
	*ROM*	4.0 ± .6 (3–5)
	**total**	**59.4 ± 22.2 (29–91)**
WOMAC	*Pain*	68.5 ± 28.8 (20–100)
	*Stiffness*	64.7 ± 29.7 (12.5–100)
	*Daily activities*	56.6 ± 25.9 (10.3–94.1)
	**total**	**59.7 ± 25.6 (15.6–93.8)**
UCLA		3.6 ± 1.5 (1–6)
EQ-5D		10.7 ± 2.6 (7–15)
EQ-5D(%)		52.1 ± 21.6 (20–90)

*AoD* absence of deformity; *EQ-5D* Euroqol-5-Dimensions; *HHS* Harris Hip Score; *ROM* range of motion; *SD* standard deviation; *UCLA* University of California, Los Angeles (score); *WOMAC* Western Ontario and McMaster’s University Osteoarthritis Index

Radiographic evaluation showed that 6 DMCs (35.3%) were fully embedded into the well-fixed cup while 11 (64.7%) were not. In twelve patients (70.6%) a higher anteversion angle of the DMC as compared to the retained metal shells was visible, while in 7 patients both implants had the same anteversion. This finding was constant in the time course between postoperative and FU radiographs. The mean inclination of the well-fixed and the new cup both postoperatively and at FU as well as the center of rotation and lateral edge analysis are displayed in Table 3. In summary, there were no differences between postoperative and FU radiographs implying a stable fixation of the DMCs.

**Table 3:** Radiographic analysis (*n* = 17) and comparison of postoperative and FU radiographs

		postoperative	FU	P
**Inclination**				
	Well-fixed cup	46.8 ± 6.2 (36.9–60.0)	47.2 ± 6.6 (36.9–57.2)	.519
	New cup	43.3 ± 9.1 (26.2–62.7)	43.7 ± 10.6 (27.0–69.8)	.594
	p	.182	.194	
	*New cup more inclined*	12/17 (70.6%)	12/17 (70.6%)	
**Center of Rotation (δ)**		2.8 ± 2.4 (.0–9.0)	2.8 ± 2.1 (.0–9.0)	1.00
**Edge (δ)**		3.4 ± 1.7 (.0–6.0)	3.2 ± 1.8 (.0–6.0)	.593
	*New cup further lateral*	14/17 (82.4%)	13/17 (76.5%)	
**Anteversion**	*New cup > well-fixed cup*	12/17 (70.6%)	12/17 (70.6%)	
**Coverage**	New cup fully covered	6/17 (35.3%)	6/17 (35.3%)	

Categorical variables are presented as counts with percentages, continuous variables as mean with standard deviation and range. A *p* value of less than .05 indicates statistical significance. FU, follow-up; δ, difference

The loose DMC showed an additional inclination of 7.5 degrees on FU radiographs when compared with the postoperative radiographs.

## Discussion

The “cup in cup” technique in combination with DMCs was shown to be a successful procedure in the short-term FU. While confirming the findings of previous comparable studies, we found that this technique may also be applied to non-elective surgery, such as in periprosthetic femoral fractures.

A first biomechanical evaluation of the double-socket technique in combination with DMCs was performed by Wegrzyn et al. Compared with conventional PE liners, the pull-out forces until failure of the construct were higher when using a cemented DMC. Analysis of the failure mechanism showed that the loosening occurred in the interface between the cement and the cementless metal cup, while the interface between the DMC and the cement remained intact [13]. These biomechanical results emphasize two main aspects. Regarding the well-integrated cementless cup, a maximal roughness of the inner surface should be achieved. Therefore, all possible screws should be removed and mechanical roughening with a burr should be performed in order to promote cement adhesion. Studies have shown a 20% increase in stability after shell roughening [4, 13, 18].

Regarding the DMC, an implant explicitly used for cemented implantation with peripheral radial and concentric circumferential grooves which oppose torsion and lever loading [10] should be used. Plummer et al. evaluated a mixed collective of patients undergoing revision THA of which a certain number were treated with a DMC cemented into a well-fixed shell [19]. While two cases of a DMC liner cementation of a modular DMC system failed, all DMCs specifically designed for cemented fixation survived [19].

Up until now, three clinical studies have been published on this topic [5–7]. Chalmers et al. [5] investigated 18 patients undergoing revision THA with a monoblock DMC construct for different reasons of which an undisclosed number had a DMC cemented into an existing well-fixed cup while the other patients underwent a full cup revision. Overall, the authors found no dissociations but three re-dislocations after a mean FU of 3 years of which one required a revision with a constrained liner while two required open reduction of the DMC. Moreta et al. [6], reporting on 10 DMCs cemented into well fixed shells, had one recurrent dislocation which was treated with resection arthroplasty due to the patient’s various comorbidities. The most recent study on this topic by Wegrzyn et al. [7] found no dislocation or dissociation of the DMC construct after a mean FU of 3.5 years in a study collective of 28 patients. This is of special importance since patients with periprosthetic fractures have a postoperative dislocation rate of over 10% which itself is associated with a heightened mortality [20].

Overall, all studies had comparable baseline patient characteristics as well as mean FU durations. Furthermore, complication rates were comparable. The main contrast to the other studies is the inclusion of non-elective cases including periprosthetic Vancouver 2B and 2C fractures which made up almost 40% of our collective. The other studies only included elective patients with liner failure or instability. During FU, all three studies showed HHS scores between 71 and 88. The fact that we also included periprosthetic fractures for the “cup in cup” technique led to additional extensive surgery on the femoral side which- taken together with high mean age at surgery (78.55 ± 7.05) as well as high preroperative ASA scores (> 80% ASA 3) in most cases- may explain the reduced functional outcomes and the higher level of mortality during FU (Table 4). A British Joint Registry- based analysis evaluating periprosthetic fractures showed an increased mortality for patients in the high-risk group (age ≥ 75, ASA ≥ 3) leading to a one-year-mortality of 15% for women and 21% for men and a five-year-mortality of 48% and 60%, respectively [21].

**Table 4:** Comparison of clinical studies on the “cup-in-cup” technique with DMCs

study	n	Age (∅)	FU (y)	comorbidities	function	dislocation (%)	dissociation (%)	time (min)	other
Chalmers [5]	18	64	3.0	/	HHS 82	3/18 (17%)	0%	/	2 PPF
Moreta [6]	10	79	3.5	CCI 4.3	HHS 71	10%	0%	/	/
Wegrzyn [7]	28	82	3.5	56/44% ASA 3/2	HHS 88	0	0	107’	/
Bellova	33	79	2.5	ASA 2.72 CCI 5.1	HHS 59 WOMAC 60	2/26 (7.7%)	1/24 (4.2%)	124′	1 infection 1 PPF

*ASA* American Society of Anesthesiologists (score); *CCI* Charlson Comorbidity Index; *FU* follow-up; *HHS* Harris Hip Score, *min* minutes; *n* number; *PPF* periprosthetic fracture; *WOMAC* Western Ontario and McMasters University Osteoarthritis Index; *y* years; *∅* average

An advantage of the double socket technique may be the reduced surgery duration in comparison with a full acetabular component exchange. The fact that the mean surgery duration of 124 min (min.) in the present study was somewhat longer compared to that reported by Wegrzyn et al. [7] (107 min.) may be attributed to the fact that some of our cases were not isolated cup revisions, but in approximately 40% of the cases combined cup and stem revisions, mostly due to periprosthetic fracture. In isolated cup revisions within our collective, the mean surgery duration was 92 min. when compared with 169 minutes for combined cup and stem revisions.

We observed one cup dissociation in our study collective shortly after surgery while none were reported in the other clinical studies. The difficulty of the double socket technique is achieving firm fixation of the inner cup. Failure can occur on the side of the fixed implant due to missing surface roughness or due to an improper cement mantle possibly providing inadequate fixation of the DMC. Wegrzyn et al. recommend a cement mantle of 2–3 mm around the DMC requiring a DMC which is – depending on the thickness of the cementless cup – at least 10 mm smaller than the outer implant diameter of the fixed cup [13]. If this recommendation is considered, cementless cups smaller than 54 mm would disqualify for the double socket technique. Since the size of cementless cups is often smaller than size 54, we accepted an eccentric positioning of DMC in several cases (65%) possibly with a thinner cement mantle. This allowed us to treat cementless cups up to a size of 48 mm with the double socket technique. As a consequence, we lateralized the center of rotation of the hip joint by about 3 mm and left the edge of the DMC uncovered by the former cup, which may lead to edge loading. In the particular case of the early cup loosening, the undercoverage as well as the absence of shell roughening could have caused the failure.

Additionally, not only were several cups lateralized, but most of them were corrected in inclination and anteversion in order to achieve a more physiological position, especially in order to avoid posterior dislocation using the posterolateral approach. Whether this method is more prone to failure cannot be fully answered with the available data. Although 16 out of 17 DMCs showed no signs of loosening at FU, the sample size of the total collective is too small and the FU time too limited to allow for a final conclusion.

In comparison to the other studies (0–17%) [5–7] we had a similar dislocation rate (7,7%). The two dislocations both occurred at the small DMC size of 43 mm. It is accepted that the smaller the DMC size, the smaller the jumping distance is until dislocation occurs [10]. This should be considered when applying the double socket technique with DMCs.

Possible discussed downsides apart from an expected increased polyethylene wear is the increased risk of taper corrosion. In a series of retrieved DMCs, Lombardo et al. found moderate fretting and corrosion damage around the trunnion as well as at the femoral head [22]. The changes were not correlated with the positioning of the implant according to the safe zones by Lewinnek et al. [23], although larger studies are needed to allow for a proper conclusion. Apart from galvanic corrosion which may occur with every modular head-taper interface, the use of a large head may lead to an increased friction at the taper. However, this could not be proven in a mathematic model with experimental validation [24]. As with conventional hip prostheses, an impingement between the taper and the cup can also lead to metal wear. In one case with a highly anteverted DMC, a beginning defect of the posterior aspect of the taper of a cementless titanium revision stem could be detected which was probably caused by posterior implant-to-implant impingement. The patient remained asymptomatic and had no dislocations at the latest FU and is closely monitored.

Our study has several limitations. First it is limited by its retrospective nature and its inherent limitations, including missing or incorrect data due to previous poor recordkeeping as well as selection, misclassification, or information bias. This includes the lack of preoperative PROMs making the evaluation of postoperative improvement difficult. The evaluation of cup positioning in plain x-rays has a certain margin of error due to varying pelvic positioning or central beam angles as well as measuring inaccuracy by the observer. Moreover, although to our knowledge being the largest study on this topic, it is still hampered by the low sample size. Although we followed up all living patients, a considerable number had died at FU. A further limitation worth discussing is the omitted shell roughening of the primary implant. Noteworthy, some of our surgeries were conducted with a small primary implant in place which also meant accepting a DMC position not in line with the primary implant. Whether this variation is more failure-prone can only be evaluated with longer FUs with lager patient cohorts, possibly in a multi-center setting.

## Conclusions

The results concerning the double socket technique presented in this study are promising, however these are merely short to mid-term results. The double socket technique in combination with DMCs is a viable “off-label” method in both elective and non-elective THA revision, especially in elderly and frail patients who benefit from a reduced operation time. When performing this procedure, a proper cementation technique, an adequate cup positioning as well as selection of a large enough DMC are crucial factors to be considered for treatment success.

## Data Availability

The datasets supporting the conclusion are available upon reasonable request.

## Abbreviations

DMC: Dual mobility cup
EQ5D: Euroqol-5-dimensions (score)
FU: Follow-up `
HHS: Harris Hip Score
PE: Polyethylene
PJI: Periprosthetic joint infection
PROM: Patient related outcome measure
THA: Total hip arthroplasty
WOMAC: Western Ontario and McMasters University Osteoarthritis Index
